# What type of relationship is learned during visual statistical learning?

**DOI:** 10.1371/journal.pone.0342272

**Published:** 2026-02-20

**Authors:** İlayda Nazlı, Floris P. de Lange

**Affiliations:** 1 Department of Psychology, Middle East Technical University, Ankara, Türkiye; 2 Donders Institute for Brain, Cognition and Behaviour, Radboud University, Nijmegen, The Netherlands; University of Hamburg, GERMANY

## Abstract

Statistical learning enables observers to extract regularities from their environment, but what statistical regularity is extracted remains debated. While previous research has mainly focused on conditional probability, recent evidence suggests that observers may instead learn the uniqueness of predictive relationships. In two visual statistical learning experiments, we manipulated the strength and uniqueness of associations between two object stimuli. We contrasted the predictions of three metrics of associative strength, which incorporate the strength and uniqueness of the association differentially. Participants viewed sequences of objects with varying transitional structures and completed an incidental categorization task. Reaction time benefits for expected versus unexpected stimuli were used to gauge learning. Across two experiments, learning benefits were best predicted by the dual factor heuristic (DFH), a heuristic that jointly considers the conditional probabilities of cue given outcome and outcome given cue. This metric predicted learning behavior better than either the conditional probability of outcome given cue, or the normative metric ΔP, which considers the difference in conditional probabilities of outcome given cue, compared to outcome given no cue. These results suggest that visual statistical learning is primarily guided by a heuristic calculation of uniqueness, as formalized by DFH, rather than either simple conditional probability or ΔP.

## Introduction

Learning is a fundamental aspect of our life, enabling us to develop and refine our internal representations of the world. A key component of this process is the ability to form associations between events that are systematically related across space and time [1]. Our environment is filled with such regularities, making it essential for us to detect recurring patterns to predict future inputs, prepare appropriate responses, and adapt flexibly to changing conditions. Observers can automatically extract these patterns from the environment over multiple exposures, even without intentional effort to learn or awareness of the learning process. This form of learning is known as statistical learning [2–6]. Statistical learning often leads to more efficient information processing, resulting in faster and more accurate behavioral responses to structured and predictable stimuli compared to unexpected ones [7–11]. On a neural level, it is typically associated with reduced neural activity for expected stimuli, reflecting an optimization of processing resources based on prior context [10,12,13].

**Fig 1:**
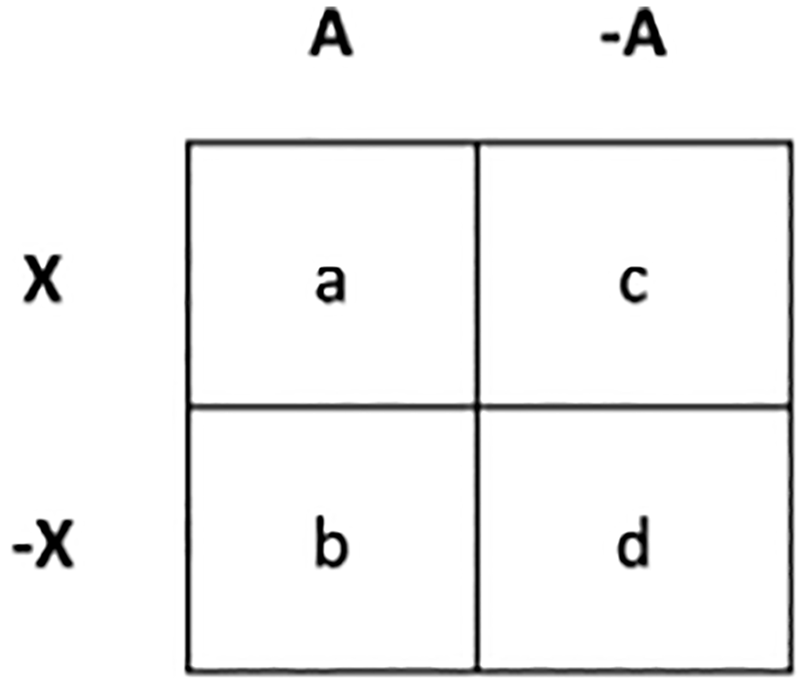
A matrix representing the relationship between event A and event X. A and ¬A respectively represent the occurrence and non-occurrence of leading stimulus A; X and ¬X respectively represent the occurrence and non-occurrence of trailing stimulus **X.**

This ability to extract regularities raises a fundamental question: What types of statistical regularities are extracted, and which metrics best govern their extraction? To address this, prior research has examined key statistical metrics that influence learning, with a particular focus on joint probability and conditional probability. Joint probability refers to the frequency with which two stimuli co-occur relative to other stimulus pairs. In contrast, conditional probability measures the likelihood of one stimulus occurring given the presence of another, capturing the strength of their association. The association between two events can be represented by 2×2 matrix as shown in Fig 1. This figure shows the association between A and X: A is followed by X. A and ¬A respectively represent the occurrence and non-occurrence of leading stimulus A, and X and ¬X respectively represent the occurrence and non-occurrence of trailing stimulus X. The letters in the cells (i.e., a, b, c, d) represent the relative frequencies of the presence and absence of A and X: a cell shows the number of ‘A is followed by X (A→X)’ observations, b cell shows the number of ‘A is followed by a different trailing stimulus (A→Y)’ observations, c cell shows the number of ‘X follows a different leading stimulus (B→X)’ observations and d cell shows the occurrence of neither A nor X (B→Y). These four cells together determine the association strength, but differently for different metrics. Joint probability is computed as $P(A,X)=\;\frac{a}{a+b+c+d}$. On the other hand, conditional probability, considers only cells a and b, measuring the likelihood of X occurring given that A has occurred (i.e., $P\left(X|A\right)=\;\frac{a}{a+b}$). Research by Fiser and Aslin (2002) demonstrated that learners primarily rely on conditional probability rather than joint probability when extracting statistical regularities from the environment. Tracking conditional probabilities allows individuals to form expectations about future occurrences, making it a crucial mechanism in statistical learning. As a result, conditional probability has been widely regarded as the dominant metric guiding the extraction of regularities, with stronger associations being more effectively learned.

Conditional probability can be limited in explaining certain situations. For instance, if A often leads to X, the conditional probability of X given A will be high, suggesting a strong relationship between A and X. As a result, observers are likely to learn the A → X association. However, if X frequently appears without A and following a stronger predictor, then the predictive power of A over X weakens. In this case, the A → X association may not be learned as strongly by observers. As a result, it might be more adaptive to ignore A and focus on the more reliable predictor instead. To assess whether A is a useful predictor of X, one should compare the probability of X occurring with A to the probability of X occurring without A [14]. This relationship is captured by ΔP [15]. According to ΔP, observers not only track the strength with which a stimulus follows another, but also track whether a stimulus uniquely predicts the other (i.e., $\Delta P=\text{P}\left(\text{X}|\text{A}\right)-\text{P}\left(\text{X}|\sim \text{A}\right)=\;\frac{a}{a+b}-\;\frac{c}{c+d}$). Therefore, in the example above where A is not a strong predictor of X, ΔP of X given A is low, potentially leading to a weak A→X association.

ΔP is commonly used in causal reasoning studies where participants are actively encouraged to learn and make judgments about the relationships between events [16]. One well-known example is the blicket detector paradigm, which is often employed to study how people intentionally learn causal relationships in order to draw accurate conclusions [17–21]. In this paradigm, participants interact with a blicket machine and are tasked with identifying which blicket cause the machine to activate.

Research using this paradigm has demonstrated that ΔP influences causal inferences in both children [21] and adults [18,19]. In contrast, other studies have observed causal learning effects in infants [22,23], who were not explicitly instructed to learn causal relationships but instead learned through passive exposure to statistical patterns. This suggests that statistical learning might be more attuned to unique predictive relationships, rather than simply relying on conditional probabilities, as some previous research has proposed [24]. In a visual statistical learning task, it was found that participants failed to grasp relationships between events with high conditional probabilities when the ΔP between them was low [25]. This indicates that statistical learning may be driven by unique predictive relationships, challenging the assumptions of earlier studies that emphasized strong relationships.

This conjecture aligns with the Dual Factor Heuristic (i.e., $DFH=\;\sqrt{P(X|A)\times P(A|X)}=\;\frac{a}{\sqrt{\left(a+b)\times (a+C\right)}}$), which emphasizes the importance of identifying unique predictive relationships to better explain how observers learn and make causal judgments [26,27]. Observers using the ΔP to form associations focus equally on both the occurrence and non-occurrence of events, processing a-b-c-d cells systematically and rationally [28–30]. However, it has been suggested that observers focus on these four cells differentially [28,31], placing greater emphasis on the occurrence of events while often ignoring the d cell [26,28]. In contrast to the more rational and analytic ΔP, observers using the DFH focus primarily on the occurrence of events, disregard the d cell, and process the relative frequencies of the presence and absence of A and X rapidly and with low effort [28–30]).

In the current study, we aimed to explore whether visual statistical learning is driven by unique predictive relationships and which metrics of uniqueness best describe this process. On each trial, participants were shown two consecutive visual objects and asked to determine whether the two objects belonged to the same category. Unbeknownst to participants, we manipulated the strength and uniqueness of the relationship between the leading and trailing objects, such that the trailing object followed either a consistent leading object most of the time, or different leading objects. While most statistical learning studies have examined the detection of regularities embedded in continuous streams of stimuli [2,4,11,31–33], the current study adopts a related but distinct approach. Some studies have instead presented two successive stimuli on each trial, with conditional probabilities controlling their pairing [3,10,13]. In terms of neural processing, both continuous streams [34] and pairs [35] show comparable modulations of sensory responses after statistical learning, suggesting that both paradigms engage similar underlying learning mechanisms. The present study adopts this latter, pair-based approach to investigate which forms of uniqueness best account for visual statistical learning. We evaluated statistical learning by presenting participants with expected and unexpected object pairs and measuring how fast they respond to the category of object pairs. Successful learning was indexed by faster reaction times to expected relative to unexpected trailing objects [9–11]. In summary, our findings suggest that observers are more sensitive to unique predictive relationships than to conditional probability, and that statistical learning appears to be governed by the Dual Factor Heuristic (DFH) rather than ΔP.

## Experiment 1

### Method

#### Participants.

The experiment was performed online by using the Gorilla platform [36], and participants were recruited through the Prolific platform (https://www.prolific.co/) between 05/12/2024 and 19/12/2024. 231 participants started the experiment. 91 of them were screened out before they finished the experiment based on a priori exclusion criteria (see section ‘Exclusion and inclusion criteria’ below) and 40 of them left the experiment before completing the tasks. While this attrition rate may appear high, it is important to bear in mind that in online experiments that are long and require strong attentional engagement, approximately half of the participants can show inattentive behavior [37,38]. Consequently, we only included participants who showed strong motivation and adequate attention to the stimuli, as required to support learning [11]. In total, 100 participants were included in the data analysis. This final number of included participants was preregistered and provided us with >80% power to detect effects that had a small to medium effect size (Cohen’s $d_{z}$ = 0.30).

All participants had normal or corrected to normal vision, normal hearing and no history of neurological or psychiatric conditions. They provided written informed consent and received financial reimbursement for their participation in the experiment. The study followed the guidelines for ethical treatment of research participants by CMO 2014/288 region Arnhem-Nijmegen, The Netherlands. All data and code used for the analyses are freely available on the Donders Repository (https://doi.org/10.34973/bv75-q239).

#### Experimental design.

**Fig 2:**
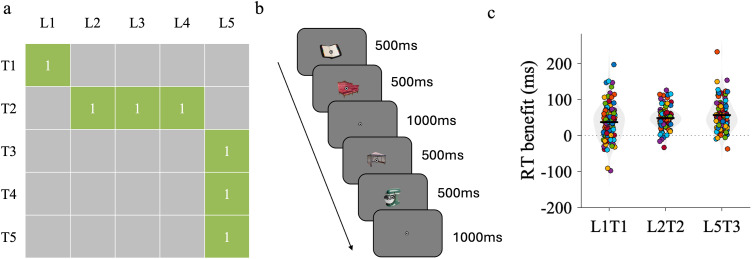
Experimental procedure and results of Experiment 1. **(a)** Statistical regularities depicted as image transition matrix with stimuli pairs in training phase. Ls represent leading stimuli, and Ts represent trailing stimuli. For clarity, only one representative object pair per condition is shown. In the actual experiment, each condition included two distinct object pairs constructed with the same statistical structure. **(b)** Trial sequence in the training and test phases. On every trial, participants saw a leading object followed by a trailing object and indicated as quickly and accurately as possible whether the two objects belonged to the same category (electronic vs. non-electronic). **(c)** Distribution of individual reaction-time benefits (Unexpected – Expected) for each condition. Each colored dot represents one participant. Gray violins depict kernel-density estimates of the data distribution (violin width ∝ probability density). The solid black horizontal lines indicate the mean, and the thin vertical lines show the 95% confidence interval around the mean. The dotted horizontal line marks zero benefit. This visualization highlights the consistency of the expectation-driven facilitation across participants and conditions.

The experimental procedure consisted of a training phase followed by a test phase (see Fig 2a). Each phase served a distinct purpose: the training phase allowed participants to incidentally learn statistical regularities between object pairs, whereas the test phase assessed learning by introducing expected and unexpected pairings.

In the training phase, object pairs were constructed to create three types of statistical associations. Leading object L1 was always followed by trailing object T1, generating L1T1 condition (i.e., CP=1,  ΔP=1 and DFH=1) which was used as a baseline condition to gauge the behavioral learning effects of strong, one-to-one, associations. Leading objects L2, L3 and L4 were followed by trailing object T2, generating L2T2 condition (i.e., CP=1,  ΔP=0.85 and DFH=0.58). Leading object L5 was followed by trailing objects T3, T4 or T5, generating L5T3 condition (i.e., CP=0.33,  ΔP=0.33 and DFH=0.58). Each statistical condition (L1T1, L2T2, and L5T3) included two distinct object pairs that shared the same transitional structure but involved different visual items. For clarity, Fig 2 illustrates only one representative pair per condition. While keeping DFH constant and varying ∆P, we aimed to determine which form of statistical uniqueness better captures visual statistical learning. Observing a stronger RT benefit in L2T2 than in L5T3 would support ∆P-based learning, whereas similar RT benefits across both would suggest DFH-driven learning.

During the training phase, only expected trials were presented (P(trailing | leading) = 1), so RT differences between expected and unexpected stimuli could not be analyzed at this stage. Participants performed an object categorization task, indicating as quickly and accurately as possible whether the leading and trailing objects belonged to the same category (electronic vs. non-electronic). RTs from this task served as the main behavioral measure. Participants were not informed about the statistical structure of the pairs, and learning was therefore incidental.

To ensure sustained attention to the leading objects, animal detection trials were interspersed in approximately 10% of categorization-task trials. In these trials, an animal image appeared as the leading stimulus, followed by a random trailing object. Participants pressed a designated key upon detecting a leading animal. These trials prevented predictable pair formation and verified attention to the leading image. From the participants’ perspective, these trials were intermixed with the categorization task and were not signaled in advance; thus, participants had to be prepared to detect an animal on any given trial.

Additionally, attention check trials were included in approximately 10% of categorization-task trials. On these trials, a brief on-screen message (e.g., “Press the left arrow key”) instructed participants to make a specific keypress, allowing vigilance monitoring (see “Exclusion and inclusion criteria”).

Stimuli were drawn from a pool of 80 everyday objects and animals [23]. Each participant viewed 20 everyday objects and 4 animals, randomly selected to minimize potential item-specific effects. A fixation point remained on the screen throughout the experiment. Object pairs were presented sequentially with no inter-stimulus interval (500 ms each) and a 1500 ms inter-trial interval. The trial order was pseudo-randomized so that successive pairs were not identical, and each pair was equally distributed over time. Thus, any difference between expected and unexpected pairings in later phases cannot be attributed to familiarity, adaptation, or trial history. The training phase began after a short practice block (using pairs not included in the main experiment). Participants completed 336 categorization-task trials (24 repetitions per pair) and 40 animal-detection or attention-check trials.

The test phase assessed whether participants had learned the predictive structure of object pairs. Each leading object from the training phase was followed either by its previously learned trailing object (expected) or by one of four unexpected trailing objects. The expected trailing object appeared four times more frequently than any individual unexpected trailing object. However, because there were four different unexpected objects, the total number of expected and unexpected trials was equal. Presenting the expected object more frequently reduced the possibility of extinction of the learned associations. Participants performed the same object categorization task as in the training phase. The test phase included 384 categorization-task trials. Stimulus presentation timing and randomization parameters were identical to those used in training.

All data were collected in a single session per participant. The session began with a familiarization phase in which participants viewed all objects and animals. Each image was presented for 500 ms and categorized (electronic, non-electronic, or animal) within 1000 ms, followed by 1000 ms of feedback showing the correct label and object name. Participants were instructed to respond as quickly and accurately as possible, and responses were allowed during the presentation of the trailing stimulus as well as during the subsequent inter-trial interval. All images were presented twice. After familiarization, participants completed the training and test phases in order where feedback was no longer provided. The object pairs presented in the training and test phases were different from the object pairs presented in the familiarization phase in order to prevent any potential confounds.

### Exclusion and inclusion criteria

The online experiment was terminated if the percentage of correct responses during object categorization was below 80% (threshold was defined based on a preliminary pilot study) in any training or test phase (see ‘Experimental design’ and Fig 1a) or if the percentage of correct responses in attention check trials was below 80% in any of the experimental phases (see section ‘Experimental design’).

Prior to the main data analysis, we discarded trials with no responses, wrong responses, or anticipated responses (i.e., response time < 200 ms). We also rejected trial outliers (response times exceeding 2 SD from mean RT of each participant) and subject outliers (participants whose RTs exceeded 2 SD from the group mean). For the accuracy analysis of the pair recognition task, we rejected trial outliers in terms of response speed (response times exceeding 2 SD from mean RT of each participant). Based on these criteria, an average of 10.1% of correct trials were excluded in Experiment 1 and 10.6% in Experiment 2. In addition, all main analyses were repeated using median reaction times without trial-level exclusions, yielding highly comparable results (see S1 File).

### Results

We did not statistically analyze the accuracy data in the test phase. This was because the categorization task was not challenging, which was supported by the performance near ceiling levels (Experiment 1: M = 94%, SD = 3.4, 95% CI [93.5, 94.8]; Experiment 2: M = 94%, SD = 3.2, 95% CI [93.4, 94.6]).

*Analysis of RT data in test phase.* We analyzed the RT data in the test phase in order to test for incidental learning of predictable stimulus transitions.

We hypothesized that, if participants incidentally learned the statistical regularities, they would respond faster to expected than to unexpected trailing objects, particularly under high-uniqueness conditions. To test this hypothesis, a 2 (Expectation: expected/unexpected) × 3 (Condition: L1T1/L2T2/L5T3 repeated measures ANOVA was conducted to examine the main effects of expectation and condition, as well as their interaction, on reaction time using JASP (see Fig 2c).

We observed a main effect of expectation (F(1,99) = 262.95, p < 0.001, η² = 0.73), indicating overall learning and a behavioral benefit of expectation reflected in faster responses. We observed significant interaction effect between expectation and condition (F(2,198) = 7.45, p < 0.001, η² = 0.07), which was driven by expectation benefits being smaller in the L1T1 condition (37 ms) than in the L2T2 (48 ms) or L5T3 (56 ms) conditions. While this lower expectation benefit for the stimuli that had the strongest association was surprising, we speculate that it might relate to the relatively larger change in stimulus predictability between training and test phase (see Discussion below). Of primary interest was the comparison between the L2T2 and L5T3 conditions, as these allowed us to explore how CP, ΔP and DFH might differentially account for visual statistical learning. A direct comparison between these conditions did not indicate a significant interaction effect between expectation and condition (F(1,99) = 3.44, p = 0.07, η² = 0.03) with learning benefits that in fact were numerically (but not significantly) larger for the condition that had lower CP/ΔP.

To make a stronger case that participants learned the L2T2 and L5T3 conditions equally well, as previously suggested by the non-significant interaction in the ANOVA, we conducted a Bayesian paired-samples t-test. This test directly compared the learning effect (RT benefit for expected vs. unexpected stimuli) across the two conditions, which were matched in DFH but differed substantially in CP and ΔP. The analysis yielded a Bayes Factor of BF₁₀ = 0.57, providing anecdotal evidence in favor of the null hypothesis that participants learned the two conditions to a similar extent. This pattern is more consistent with DFH-based learning than with accounts emphasizing ΔP or CP, given that DFH was held constant across conditions.

### Discussion

In Experiment 1, while keeping DFH constant we varied ΔP to understand which forms of uniqueness influence visual statistical learning. Participants showed comparable learning effects for L5T3 (i.e., CP=0.33,  ΔP=0.33 and DFH=0.58) and L2T2 (i.e., CP=1,  ΔP=0.85 and DFH=0.58) pairs (i.e., 48 ms RT benefit in L5T3 and 56 ms RT benefit in L2T2). This pattern is more consistent with the notion that DFH, rather than conditional probability or ΔP, may play a predominant role in the incidental learning of object pair regularities, since participants showed similar learning for pairs that differed substantially in CP and ΔP but shared the same DFH value. It should be noted, however, that this interpretation relies on the absence of a significant difference and therefore provides only limited evidence. To support this equivalence more directly, we conducted a Bayesian paired-samples *t*-test comparing the learning effects between the L2T2 and L5T3 conditions. The resulting Bayes Factor (BF₁₀ = 0.57) provided anecdotal evidence in favor of the null hypothesis, consistent with the idea that learning may be better captured by DFH than by CP or ΔP. To further examine this possibility, we conducted a second experiment with a refined design in which ΔP and DFH were modulated in opposite directions, allowing a more direct comparison between the two metrics.

## Experiment 2

### Method

#### Participants.

The experiment was performed online by using the Gorilla platform [36], and participants were recruited through the Prolific platform (https://www.prolific.co/) between 07/02/2025 and 16/03/2025. 201 participants started the experiment. 81 of them were screened out before they finished the experiment based on a priori exclusion criteria (see section ‘Exclusion and inclusion criteria’ below) and 20 of them left experiment before completing the tasks. As a result, 100 participants were included in the data analysis, as in Experiment 1.

All participants had normal or corrected to normal vision, normal hearing and no history of neurological or psychiatric conditions. They provided written informed consent and received financial reimbursement for their participation in the experiment. The study followed the guidelines for ethical treatment of research participants by CMO 2014/288 region Arnhem-Nijmegen, The Netherlands.

#### Experimental design.

**Fig 3:**
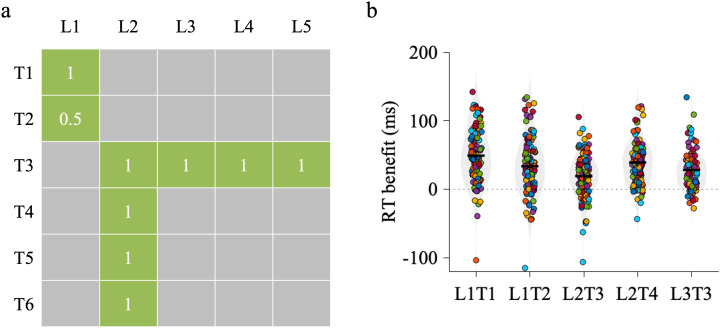
Experimental procedure and results of Experiment 2. **(a)** Statistical regularities depicted as image transition matrix with stimuli pairs in training phase. Ls represent leading stimuli, and Ts represent trailing stimuli. For clarity, only one representative object pair per condition is shown. In the actual experiment, each condition included two distinct object pairs constructed with the same statistical structure. **(b)** RT benefit (Unexpected – Expected) distributions for each condition. Colored dots represent individual participants, gray violins indicate the kernel-density estimate of the distribution, and black horizontal and vertical lines denote the mean and its 95% confidence interval, respectively. The dotted horizontal line marks zero benefit. The pattern of results demonstrates a reliable expectation effect across conditions, with stronger facilitation in conditions predicted by DFH rather than by CP or ΔP, highlighting DFH’s central role in visual statistical learning.

Experiment 2 was designed to extend the findings of Experiment 1 by more directly testing which form of uniqueness better accounts for statistical learning. To achieve this, we introduced novel pairings with more extreme transitional probabilities and controlled contrasts. Of critical importance, we aimed to distinguish between DFH and ΔP by contrasting conditions that had a positive difference in one, but a negative difference, in the other metric. The specific structure of transitions was as follows (see Fig 3a): Leading object L1 was followed by trailing objects T1, generating L1T1 condition (i.e., CP=0.67,  ΔP=0.67 and DFH=0.82). Leading object L1 was also followed by trailing objects T2, generating L1T2 condition (i.e., CP=0.33,  ΔP=0.33 and DFH=0.58). The amount of exposure to L1T2 were 0.5 times lower than that of the other object pairs. Leading object L2 was followed by trailing objects T3 which generated L2T3 condition (i.e., CP=0.25,  ΔP=0.02 and DFH=0.25) and was followed by T4, T5 or T6 which generated L2T4 condition (i.e., CP=0.25,  ΔP=0.25 and DFH=0.50). Leading objects L3, L4 and L5 were followed by trailing object T3, generating L3T3 condition (i.e., CP=1,  ΔP=0.81 and DFH=0.50). Note that some conditions shared the same set of unexpected trailing objects (i.e., L1T1 with L1T2, and L2T3 with L2T4). This design choice was intentional due to the limited number of available trailing stimuli. The same unexpected objects were used across these condition pairs to balance response mappings (i.e., ensuring equal numbers of same vs. different category responses for expected and unexpected trials). Consequently, the RT pattern for unexpected trials appears relatively stable across these conditions because of a feature inherent to the experimental design rather than a confound.

### Results

*Analysis of RT data in test phase.* We performed a 2 (Expectation: expected/unexpected) × 4 (Condition: L1T1/L2T3/L2T4/ L3T3) repeated measures ANOVA to examine which metrics (i.e., CP,ΔP and DFH) might best account for visual statistical learning (see Fig 3b). We observed main effect of expectation (F(1,99) = 261.94, p < 0.001, η² = 0.73), indicating overall learning and a behavioral benefit of expectation reflected in faster responses. We observed significant interaction effect between expectation and condition (F(3,297) = 17.26, p < 0.001, η²ₚ = 0.15). To test the role of CP,ΔP and DFH on visual statistical learning, a series of post-hoc comparisons were conducted between key experimental conditions.

As a sanity check, we first compared the L1T1 (i.e., CP=0.67,  ΔP=0.67 and DFH=0.82) and L2T3 conditions (i.e., CP=0.25,  ΔP=0.02 and DFH=0.25), which differed simultaneously in all three metrics (CP, ∆P, and DFH). While this comparison does not allow for a clean dissociation between metrics, it is included in the analysis because it represents the largest contrast in association strength across all conditions. Indeed, we observed a significant interaction effect expectation and condition (F(1,99) = 32.07, p < 0.001, η² = 0.25, significant after Bonferroni correction (adjusted α = 0.0167), 19 ms RT benefit in L2T3 and 49 ms RT benefit in L1T1). Consistent with the frequentist result, a Bayesian paired-samples t-test revealed decisive evidence for a difference between conditions (BF₁₀ = 8.55 × 10⁴), further confirming that participants were highly sensitive to large differences in statistical structure. This comparison provides a useful baseline for evaluating more targeted contrasts in subsequent analyses.

We then focused on a more subtle and informative contrast between the L1T1 (i.e., CP=0.67,  ΔP=0.67, DFH=0.82) and L3T3 conditions (i.e., CP=1,  ΔP=0.81, DFH=0.50). This comparison was particularly informative for exploring the relative contribution of ΔP and DFH, as it involved one condition with higher ΔP (i.e., L3T3) and another with higher DFH (i.e., L1T1), thereby allowing for a direct comparison between ΔP and DFH. The metrics behave differently in these cases, because DFH is equally sensitive to forward and backward conditional probabilities, whereas ΔP specifically computes the difference between two forward conditional probabilities. Again, we observed a significant interaction effect between expectation and condition (F(1,99) = 23.74, p < 0.001, η²ₚ = 0.19, significant after Bonferroni correction (p < 0.0167), 49 ms RT benefit in L1T1 and 28 ms RT benefit in L3T3). Consistent with this, a Bayesian paired-samples t-test revealed decisive evidence for a difference between the two conditions (BF₁₀ = 3.57 × 10³), providing converging support that learning effects are better explained by DFH than by ∆P.

Because one of the conditions (L1T2) was intentionally presented at half the exposure of the others, we did not include it in the same model as the equal-exposure conditions. Combining all five conditions into a single 2 × 5 ANOVA would have resulted in an unbalanced design, conflating the effects of exposure with differences in statistical metrics. To avoid this confound, we conducted separate analyses to explore the role of exposure in statistical learning, we performed a 2 (Expectation: expected/unexpected) × 2 (Condition: L1T2/L2T4) repeated measures ANOVA. These two conditions were selected because they share similar values for CP, DFH, and ∆P (i.e., L1T2: CP = 0.33, ∆P = 0.33 and DFH = 0.58 and L2T4: CP = 0.25,∆P = 0.25 and DFH = 0.50). The key difference between the two conditions lies in the amount of exposure to the stimulus, with L1T2 being presented less frequently than L2T4. This comparison allows us to examine the impact of exposure on participants’ reaction times, isolating its effect while controlling for the other factors (i.e., CP, DFH, and ∆P). We did not observe significant interaction effect expectation and condition (F(1,99) = 1.47, p = 0.23, η²ₚ = 0.02, 33 ms RT benefit in L1T2 and 39 ms RT benefit in L2T4), suggesting that the uniqueness of object pairs may play a more critical role than exposure frequency in statistical learning.

### Discussion

In Experiment 2, we further explored the role of DFH and ΔP by directly comparing them against each other. We manipulated DFH and ΔP in the opposite directions to create a more nuanced design matrix. This allowed us to investigate the relative contributions of these metrics to statistical learning and determine whether DFH or ΔP influence learning. The results from the Experiment 2 suggest that DFH may play a predominant role in visual statistical learning. The most informative comparison in Experiment 2 involved L1T1 and L3T3 conditions, which were diverged in opposite directions in ΔP and DFH. While ΔP was higher in the L3T3 condition, participants showed a greater reaction time benefit for the L1T1 condition, which had a higher DFH value. This pattern appears more consistent with DFH-based learning than with accounts emphasizing ΔP. An additional comparison between L1T1 vs. L2T3 shows that larger differences in DFH contributed to a more substantial reaction time benefit in L1T1, further suggesting that DFH may have a stronger influence on statistical learning than CP or ΔP. Lastly, the comparison between L1T2 and L2T4, which was designed to isolate the effect of exposure, did not show a significant effect, suggesting that DFH is more influential in statistical learning than the amount of exposure to the stimuli. Taken together, these findings indicate that DFH provides the most consistent account of the observed learning effects.

## General discussion

Statistical learning allows individuals to optimize their behavior by efficiently utilizing limited cognitive resources to detect recurring patterns in the environment. A key question, however, is what kind of structure is learned during this process. Previous research has shown that observers typically learn the strong predictive relationships between events or CP. However, a recent study by Leshinskaya and Thompson-Schill [25] suggests that observers may focus more on the unique predictive relationships between events, rather than just their conditional probability. The current study aims to further explore how the unique predictiveness of stimuli influences statistical learning, specifically examining which forms of uniqueness are utilized during the learning process. We investigated the relative contributions of CP, DFH, and ΔP in visual statistical learning. Our findings offer new insights into the type of predictive relationship that is extracted from the environment, suggesting that DFH may play a predominant role in visual statistical learning, while CP and ΔP show limited explanatory value in this context.

The results of Experiment 1 and Experiment 2 provide converging evidence that DFH provides the most consistent account for visual statistical learning, surpassing the effects of CP and ΔP. In Experiment 1, participants learned the pairs with a CP of 0.33 as effectively as those with a CP of 1, suggesting that uniqueness, rather than conditional probability, may play a more significant role in automatic learning of object pair regularities. Furthermore, participants showed similar learning for pairs with ΔP values of 0.33 and 0.85. This pattern appears more consistent with DFH-based learning than with accounts emphasizing ΔP. Experiment 2 directly contrasted DFH and ΔP by creating conditions varying DFH and ΔP in opposite directions. The findings indicated that higher DFH values were generally associated with stronger learning effects, as measured by reaction time benefits. Additionally, the amount of exposure did not significantly affect reaction times when DFH is strong enough to learn the relationship, further suggesting that DFH may exert a stronger influence on statistical learning than exposure frequency. These findings underscore the potential importance of DFH in shaping how individuals extract regularities from their environment, with CP and ΔP playing a less role in visual statistical learning.

Our findings build upon and extend the work of Leshinskaya and Thompson-Schill [25], who suggested that statistical learning may be more sensitive to the uniqueness of predictive associations than to conditional probabilities. In their study, they found that learning was better for associations where ΔP was higher. However, their conclusions were based primarily on ΔP, and they did not consider alternative metrics such as DFH that capture uniqueness in a different computational form. In contrast, our study systematically compared CP with ΔP and DFH across multiple conditions that dissociate these metrics. By doing so, we not only replicate the general finding that learning is sensitive to uniqueness, but also find that DFH appears to be a stronger predictor than ΔP. Our results therefore refine and extend the uniqueness account by providing a more precise computational characterization of the learning mechanism.

While Ramachandran et al. [39] did not explicitly discuss DFH, their findings provide compelling neural evidence for the importance of bidirectional contingency in learning visual transitions. In their study, monkeys were trained with image pairs that varied systematically in their conditional probabilities: in the 1:1 condition, each leading image predicted and was predicted by the same trailing image (P(B|A)=1, P(A|B)=1); in the 1:2 and 2:1 conditions, only one of these conditional probabilities was reduced to 0.5. Neural recordings from inferotemporal cortex revealed stronger prediction suppression for the 1:1 condition than for both 1:2 and 2:1, with no difference between the latter two. This symmetrical pattern indicates that prediction suppression depends on the mutual contingency between stimuli rather than on a single directional conditional probability. Such findings align closely with the present behavioral results, in which learning strength was best explained by DFH, a bidirectional measure that integrates both forward and backward predictive relations. From a broader theoretical perspective, this interpretation also converges with chunk-based accounts of statistical learning [40], which propose that learners encode co-occurring elements as integrated units rather than computing isolated transitional probabilities. DFH’s bidirectional formulation may thus provide a computational link between neural evidence for contingency-based coding and cognitive theories emphasizing holistic chunk formation.

The present study relied on reaction times as a behavioral index of statistical learning, as RT provides a sensitive measure of implicit learning that can be observed even when participants have no explicit awareness of the underlying regularities (e.g., contextual cueing). More explicit tasks (e.g., judging which stimuli occurred closer together in time) might underestimate learning effects, especially when learning operates implicitly. Nevertheless, examining how different task demands or response measures influence the expression of statistical learning represents an interesting direction for future research. Furthermore, while the present results suggest that DFH provides the most consistent account of the observed learning effects, this does not preclude the contribution of other statistical metrics. Learning is likely multifaceted, and different cues (e.g., CP, ΔP, or DFH) may be prioritized under different task contexts or by different individuals. Recent findings support this view, showing that both individual and neural differences shape how statistical regularities are extracted and represented [41,42]. DFH may best capture the relative magnitude of learning effects in the current design, but other metrics might become more relevant when the task structure or learning goals change. Future work could explore how such contextual or individual factors shape the weighting of different statistical cues during learning.

In conclusion, the results of our study suggest that DFH may play a key factor in visual statistical learning, providing a more consistent account of learning behavior than CP or ΔP. Our findings indicate that observers may be particularly sensitive to the unique predictive relationships captured by DFH. Both Experiment 1 and Experiment 2 showed that learning patterns aligned more closely with DFH, even when CP and ΔP varied substantially. Together, these findings contribute to our understanding of the cognitive computations underlying statistical learning and underscore the potential relevance of bidirectional uniqueness in the detection of environmental regularities. Future work should investigate the generalizability of DFH across different tasks, domains, and sensory modalities.

## Supporting information

S1 File Alternative reaction time preprocessing. (DOCX)
