# Supplementary material for: What type of relationship is learned during visual statistical learning?
Source: PLoS One. 2026 Feb 20;21(2):e0342272. doi: 10.1371/journal.pone.0342272 (PMC12923146; doi:10.1371/journal.pone.0342272)
Supplement: S1 File — (DOCX) [file pone.0342272.s001.docx]

**Supporting Information**

In the main analyses, reaction times were preprocessed using standard trial- and participant-level exclusion criteria, as described in the main manuscript. Based on these criteria, an average of 10.1% of correct trials were excluded in Experiment 1 and 10.6% in Experiment 2.

To assess whether the reported findings depend on the specific preprocessing procedure used in the main analyses, we conducted an additional set of analyses using an alternative approach based on median reaction times without applying trial-level exclusions. This approach is less sensitive to long-tailed RT distributions and occasional attentional lapses. The results of these analyses are reported below.

**Experiment 1**

We repeated the analyses using an alternative reaction time preprocessing approach based on median reaction times without trial-level exclusions. The overall pattern of expectation benefits across conditions was highly similar to that observed in the main analysis. Specifically, the reaction time benefit (unexpected – expected) was 41 ms in the L1T1 condition, 48 ms in the L2T2 condition, and 62 ms in the L5T3 condition. This closely mirrors the pattern reported in the main manuscript (L1T1: 37 ms, L2T2: 48 ms, L5T3: 56 ms), with expectation benefits increasing as stimulus uniqueness increased.

We first conducted a 2 (Expectation: expected vs. unexpected) × 3 (Condition: L1T1, L2T2, L5T3) repeated-measures ANOVA on reaction times. As in the main analysis, this alternative RT preprocessing analysis revealed a strong main effect of expectation, indicating faster responses to expected than unexpected trailing objects (F(1,99) = 220.53, p < .001, η²ₚ = .69). We also observed a significant Expectation × Condition interaction (F(2,198) = 5.41, p = .005, η²ₚ = .052), reflecting differential expectation benefits across conditions.

Given the theoretical focus on distinguishing between ΔP and DFH, we next examined the focused comparison between the L2T2 and L5T3 conditions. The alternative RT preprocessing analysis again showed a significant main effect of expectation (F(1,99) = 216.73, p < .001, η²ₚ = .686) and a significant Expectation × Condition interaction (F(1,99) = 5.42, p = .022, η²ₚ = .052). Consistent with the main analysis, the expectation benefit was larger in the L5T3 condition than in the L2T2 condition.

**Experiment 2**

We repeated the analyses using an alternative reaction time preprocessing approach based on median reaction times without trial-level exclusions. The overall pattern of expectation benefits across conditions was again highly similar to that observed in the main analysis. Specifically, the reaction time benefit (unexpected – expected) was 56 ms in the L1T1 condition, 14 ms in the L2T3 condition, 32 ms in the L3T3 condition, 37 ms in the L1T2 condition, and 44 ms in the L2T4 condition. This closely mirrors the pattern reported in the main manuscript (L1T1: 49 ms, L2T3: 19 ms, L3T3: 28 ms, L1T2: 33 ms, L2T4: 39 ms), with only minor numerical differences across preprocessing approaches.

We first conducted a 2 (Expectation: expected vs. unexpected) × 4 (Condition: L1T1, L2T3, L2T4, L3T3) repeated-measures ANOVA on reaction times. As in the main analysis, this alternative RT preprocessing analysis revealed a strong main effect of expectation, indicating faster responses to expected than unexpected trailing objects (F(1,99) = 280.03, p < .001, η²ₚ = .739). We also observed a significant Expectation × Condition interaction (F(3,297) = 20.52, p < .001, η²ₚ = .172), reflecting differential expectation benefits across conditions. This pattern closely mirrors the results reported in the main manuscript.

As a sanity check, we first compared the L1T1 and L2T3 conditions, which differ simultaneously in CP, ΔP, and DFH and therefore represent the largest contrast in association strength across conditions. As in the main analysis, the alternative RT preprocessing analysis revealed a significant Expectation × Condition interaction (F(1,99) = 37.21, p < .001, η²ₚ = .273), indicating a larger expectation benefit in the L1T1 condition than in the L2T3 condition. This pattern closely mirrors the results reported in the main manuscript and confirms that participants were highly sensitive to large differences in statistical structure.

We next focused on the contrast between the L1T1 and L3T3 conditions, which provides a more subtle test of the relative contributions of ΔP and DFH, as the two metrics diverge across these conditions. As in the main analysis, the alternative RT preprocessing analysis revealed a significant Expectation × Condition interaction (F(1,99) = 20.85, p < .001, η²ₚ = .174), with a larger expectation benefit in the L1T1 condition than in the L3T3 condition. This pattern closely mirrors the results reported in the main manuscript and supports the conclusion that learning effects are better captured by DFH than by ΔP.

We next compared the L1T2 and L2T4 conditions, which differ in exposure frequency while being closely matched in CP, ΔP, and DFH. In the alternative RT preprocessing analysis, we observed a small Expectation × Condition interaction (F(1,99) = 4.27, p = .041, η²ₚ = .041), reflecting numerically similar expectation benefits in the two conditions (L1T2: 37 ms; L2T4: 44 ms). Given the small effect size and the close similarity in reaction time benefits, this result provides only limited evidence for an effect of exposure frequency and is broadly consistent with the conclusion drawn in the main analysis.
